# Feasibility of Using Microsoft Kinect to Assess Upper Limb Movement in Type III Spinal Muscular Atrophy Patients

**DOI:** 10.1371/journal.pone.0170472

**Published:** 2017-01-25

**Authors:** Xing Chen, Juliane Siebourg-Polster, Detlef Wolf, Christian Czech, Ulrike Bonati, Dirk Fischer, Omar Khwaja, Martin Strahm

**Affiliations:** 1 Data Science, Roche Pharmaceutical Research and Early Development Informatics, Roche Innovation Center Basel, F. Hoffmann-La Roche, Ltd., Basel, Switzerland; 2 Translational Technologies and Bioinformatics, Pharmaceutical Sciences, Roche Pharmaceutical Research and Early Development, Roche Innovation Center Basel, F. Hoffmann-La Roche, Ltd., Basel, Switzerland; 3 Biomarker Experimental Medicine, Neuroscience, Roche Pharmaceutical Research and Early Development, Roche Innovation Center Basel, F. Hoffmann-La Roche, Ltd., Basel, Switzerland; 4 Division of Neuropediatrics, University of Basel Children’s Hospital, Basel, Switzerland; 5 Department of Neurology, University of Basel Hospital, Basel, Switzerland; 6 Translational Medicine, Neuroscience and Rare Diseases, Roche Pharmaceutical Research and Early Development, Roche Innovation Center Basel, F. Hoffmann-La Roche, Ltd., Basel, Switzerland; University of Edinburgh, UNITED KINGDOM

## Abstract

Although functional rating scales are being used increasingly as primary outcome measures in spinal muscular atrophy (SMA), sensitive and objective assessment of early-stage disease progression and drug efficacy remains challenging. We have developed a game based on the Microsoft Kinect sensor, specifically designed to measure active upper limb movement. An explorative study was conducted to determine the feasibility of this new tool in 18 ambulant SMA type III patients and 19 age- and gender-matched healthy controls. Upper limb movement was analysed elaborately through derived features such as elbow flexion and extension angles, arm lifting angle, velocity and acceleration. No significant differences were found in the active range of motion between ambulant SMA type III patients and controls. Hand velocity was found to be different but further validation is necessary. This study presents an important step in the process of designing and handling digital biomarkers as complementary outcome measures for clinical trials.

## Introduction

Since the introduction of the Microsoft Kinect sensor in 2010 together with its software development kit (SDK), its value as a low cost, portable and marker-free motion capture system has been widely examined. Through its depth sensor and software application programming interface (API), three-dimensional movement is tracked and locations of 20 body points, composing a skeletal model of the user, are output at a frequency up to 30 Hz. Though originally developed for gaming purposes, other fields of application of the Kinect sensor such as gait analysis [1, 2], energy expenditure [3], muscle functions [4, 5] and rehabilitation [6, 7] have been studied. Repeatability, reliability and validity of Kinect’s measurements have been investigated for healthy subjects [8–10] and in different diseases such as stroke [6, 11], Parkinson’s disease [7, 12, 13] and muscle dystrophinopathy [4, 5, 14].

Spinal muscular atrophy (SMA) is an autosomal recessive neuromuscular disease characterized by degeneration of motor neurons in the spinal cord and resulting in muscle atrophy and varying degree of weakness [15]. The severity is highly variable and patients with heterogeneous clinical features are classified into three phenotypes on the basis of age of onset and maximum achieved motor function [15, 16]. Type I SMA infants never sit independently. Type II SMA children sit at some point during their childhood, but never stand or walk independently. Type III SMA children and adults are able to stand and walk independently at some point in their childhood.

Functional rating scales provide clinically meaningful information on the course of the disease [17–20] and are used increasingly as primary outcome measures to ultimately judge the disease progression and drug efficacy. Many different scales have been used to assess different functional aspects related to daily living activities in SMA natural history studies and clinical trials. The Hammersmith Functional Motor Scale (HFMS) [20] and the Motor Function Measure (MFM) [21] are the most widely referred and validated. But the broad clinical spectrum of SMA patients makes the motor function assessment challenging. A longitudinal multi centric study has shown that the HMFS appeared to be more suitable in strong non ambulant patients while the MFM appeared to be more sensitive to the changes in the very weak patients [17]. Test of Infant Motor Performance Screening Items (TIMPSI) [22] and Children’s Hospital of Philadelphia Infant Test of Neuromuscular Disorder (CHOP INTEND) [23] are developed for type I infants. At the other end Six-Minute Walk Test (6MWT) [24] is adopted to measure lower limb fatigue for ambulant type III patients. Upper Limb Module (ULM) [25] is specifically designed to measure upper limb function in non-ambulant patients. Other scales such as the Egen Klassifikation Scale v.2 (EK2) [26] and Gross Motor Function Measure (GMFM) [27] also show potential in capturing different aspects of muscle weakness. Combinations of different rating scales have been used to cover the wider spectrum of the disease. An expanded version of HFMS [28] is developed by adding 13 GMFM items allowing for evaluation of ambulant SMA patients. Another example is SMA functional composite score [29] which is constructed based on the first principal component of HFMSE, ULM and 6MWT. In order to evaluate the psychometric properties of those clinical rating scales, Rasch methodology was applied to nine functional rating scales [18] and identified several issues impacting their validity. Psychometric weaknesses for all scales were revealed as clinicians interpret and score the children inconsistently. Another problematic feature for some of scales is that several sub-scores, addressing different aspects of the disease, are joined into a total score. This makes interpretation of these scores difficult.

The ability to assess muscle dystrophinopathy with the Kinect has been explored by measuring the 3-dimensional reachable area of the upper body [4, 14]. However, in these two studies only the volume reachable by hand for a patient was measured without analysing specific arm limitations. It was shown that in SMA patients the reduced range for joint motion in shoulder, elbow, wrist, hip, knee and ankle were positively correlated with functional scales [30, 31]. Such values, previously recorded with transparent goniometers [30] can now be easily obtained and analysed using the body points locations recorded by the Kinect sensors. To obtain detailed and objective outcome measures for upper body movement, we developed a Kinect game specifically designed to capture limitations in active joint motion and the game was adopted in a clinical study to determine the feasibility of using the Microsoft Kinect sensor to rate patients with spinal muscular atrophy.

## Methods

### Game design and implementation

**Fig 1:**
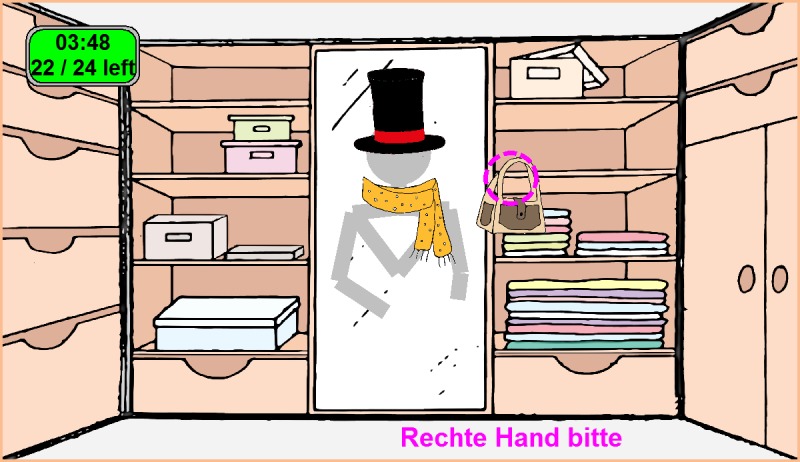
Game scene. In the game scene, a visual skeleton figure represents the body of the subject. A flashing indicator and information below (pink) instruct the subject where to reach with which hand. On the upper left corner a counter and a timer are shown.

Based on the understanding that SMA patients have limited joint range of motion, a prototype game targeting the upper limb function was designed. In a cartoon scene of a wardrobe background, ten virtual objects (e.g. hats, scarfs, glasses, bags and birds) are displayed one after another at 5 different heights and symmetrically on both left and right side. The objects are placed according to the subject’s anthropometric measures as determined by Kinect sensors. The participant is instructed to extend the elbow while raising the arm to get the object. After the object is reached by the hand, the participant is further instructed to flex the elbow in order to place the object on the virtual body (e.g. head, neck, nose and shoulders respectively) on screen (Fig 1). The whole task is repeated once and in total 20 objects are to be picked up. In case an object is not picked up within 12 seconds, the game automatically proceeds to the next item. To provide this user-friendly gaming interface which is appealing to both children and adults as well as to retrieve skeleton information from the Microsoft Kinect sensors API, Java programming language was used. The three-dimensional coordinates of 9 upper body points as defined by Kinect sensor (head, neck, shoulders, elbows, hands and torso) of the subject were recorded over the whole course of the game and used to derive quantitative movement features such as angular range of motion (extension and flexion angles of elbows, lifting angles of arms), speed of motion, reachable spatial projection into a two-dimensional plane, etc. These data were analysed to reflect the upper limb function in SMA patients and healthy controls.

### Clinical study

The Kinect motor function assessment game was tested in an observational, single center, longitudinal study on ambulant SMA patients and controls (NCT02044029, https://clinicaltrials.gov/show/NCT02044029). All the tests were conducted by a clinician at the hospital. 18 ambulant SMA type III patients and 19 age- and gender- matched healthy controls were recruited at University Children’s hospital of Basel (UKBB). The subjects were at least 10 years old at the time of screening. The protocol was approved by the local ethics committee: Ethikomission beider Basel. All procedures were conducted according to the principles expressed in the Declaration of Helsinki. Written informed consent and compliance with the study protocol according to International Conference on Harmonisation (ICH) and local regulations were provided by all the subjects. For children below the age of legal consent, a legally authorized representative which was one of the parents was able to consent for the patient according to ICH and local regulations.

This observational study was chosen to test the developed Kinect motor assessment tool for two reasons: 1, as a first attempt to demonstrate the technical and operational feasibility, this already planned study is the closest in both timeline and location; 2, even though upper limb impairment in ambulant SMA patients is much less common, it is still worth to see if early stage progression or minor differences in high functioning patient groups can be detected from the detailed tracking of active upper limb movement, which might be a valuable complementarity to the rating scales.

Each subject participated in four testing sessions, with each session done on a separate day: at baseline, week 12, week 24 and week 48. Our Kinect-based motor task game was implemented as an exploratory tool for both patients and healthy controls. The Kinect test was performed twice in controls to assess test-retest repeatability with a resting time of about one minute. It was not performed twice in SMA patients with the intention of not causing too much burden.

The Kinect camera was set up on a flat table connected to an All-in-one screen. The subjects were comfortably seated in a chair without arm rests, about 2 meters away from the camera. The whole Kinect test lasted less than 5 minutes.

### Data handling

#### 1, Data acquisition, transfer and quality check

The three dimensional coordinates of 9 upper body points were recorded throughout the game with a time stamp at about 60 ms intervals provided by the Kinect sensor. To maintain the confidentiality standards, each subject was assigned a unique subject identification number entered into the system by the clinician. The data was transferred via internet cloud in an encrypted way. Data was checked for completeness of the test using a simultaneously recorded log file, containing validity of execution flags and correct number of performances.

#### 2, Feature extraction and filtering

27 summary movement features were extracted from the time series of coordinates. First, elbow angles, arm lifting angles, hand velocity and acceleration, motion path length, body points distances, trunk movement and reachable spatial projection were calculated for all time points. Elbow angle is defined as the angle between two 3D vectors connecting hand and elbow, elbow and shoulder respectively. Arm lifting angle is the angle of elbow point relative to the vertical axis. Hand azimuth velocity and acceleration are calculated in the spherical coordinate system [32], using the locations of shoulders, hip and hand as well as recorded time stamps. Motion path length is the total path length of the hand for the whole test. Body points’ distance is the distance of two related but not directly connected body points such as hand and shoulder. Trunk movement is defined as the deviation of the upper body from its average position, calculated by averaging across torso, shoulders, head and neck. Reachable surface is the maximal area of the 2D plane reached by the hands during the task. Secondly, the extracted features were summarized across time by median value, several quantile values, maximal and minimal value and standard deviation. Constant features were filtered out, for example minimal velocity and minimal acceleration of hand. It is noteworthy that only the data was only extracted from the active side performing the task. Total time used to complete the test was calculated for each assessment and compared across the four visits as an indicator for learning effect.

#### 3, Statistical analysis

Statistical analysis was performed using R.

PCA. In a multivariate approach a principal component analysis on all extracted features is performed to reveal overall patterns in the data and identify outlier samples.

Correlation. For all control samples two measurements per session are available. Per person the correlation of the two assessments is calculated. Repeatability is analysed through correlation coefficients and Bland-Altman plots. This is compared to the between session correlation for the control group and the patients.

ANOVA. Analysis of variance (ANOVA) is used to analyse the difference in total time spent in finishing the test to reveal the existence of learning effect.

Disease Status Model. For modelling only the first measurement per visit is used for each of the controls. Each model is fitted for all 75 features. A linear mixed effects (LME) model is fitted for each KINECT feature with CLASS (SMA / Control) as main predictor, using age, sex, BMI and height as covariates.

Feature∼CLASS+AGE + SEX + BMI + HEIGHT

For all terms the p values are extracted and FDR corrected.

All data including extracted feature files and raw data files (S1 Dataset), all code including prototype game in Java, analysis code in R and data extraction code in Python (S1 Code) as well as additional supplemental results (S1 Text) are uploaded as supplemental materials and thus publically available.

## Results

### Feasibility of implementation

All patients and controls completed the study. The acceptance of the Kinect game was very high and it was enjoyable for both patients and controls. Patients with motor impairment did not feel discouraged. They also enjoyed the instant feedback by looking at the screen. The setup was easy to handle and the only one training session was sufficient for clinicians to perform the test correctly.

### Clinical features

The demographic features are presented in Table 1. No significant difference (two sample t-test) was observed among the seven vital signs except for heights between SMA patients and healthy controls.

**Table 1:** Demographics of SMA patients and healthy controls.

	SMA	Control
**Number**	18 (13 M, 5 F)	19 (13 M, 6 F)
**Age**	32.3 ± 12.7	33.2 ± 13.9
**Weight** * **(kg)**	65.4 ± 11.1	74.7 ± 15.5
**Height** *^#^ **(cm)**	174.9 ± 11.4	175.6 ± 10.6
**BMI** ^a^* **(kg/m**^**2**^**)**	21.3 ± 2.7	24.0 ± 4.0

^a^ Body Mass Index

* Average value of one screening and 4 sessions

^#^ p < 0.05 between SMA patients and healthy controls

### Data transfer and validity check

Data was recorded, smoothly transferred via cloud and decrypted. For patients, 66 valid records out of 72 (18 patients, 4 sessions each) were used for further analysis. One removed record was incomplete, and five records were missing since the task was not performed in that session. For healthy controls, 121 valid records out of 152 (19 controls, 4 sessions, twice per session) were obtained. Two removed records were incomplete, and another five records were removed due to inconsistent measurement dates with the clinical reports. The remaining were missing since some controls were tested only once in the same session.

### Exploratory analysis

**Fig 2:**
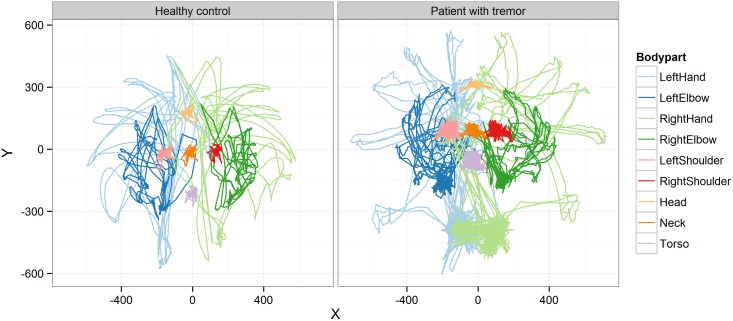
Trace plot. Movement trajectories of all 9 tracked body points in x-y dimension for a patient with a tremor and a healthy control.

Fig 2 shows an X-Y trace plot of a patient with impaired coordination and a healthy control, in which X and Y coordinate trajectories of all the 9 body points from upper body were plotted for the whole test. PCA was performed on all features derived from coordinate data, and two detected outlier samples were removed from further analysis (see discussion and S1 Text).

**Fig 3:**
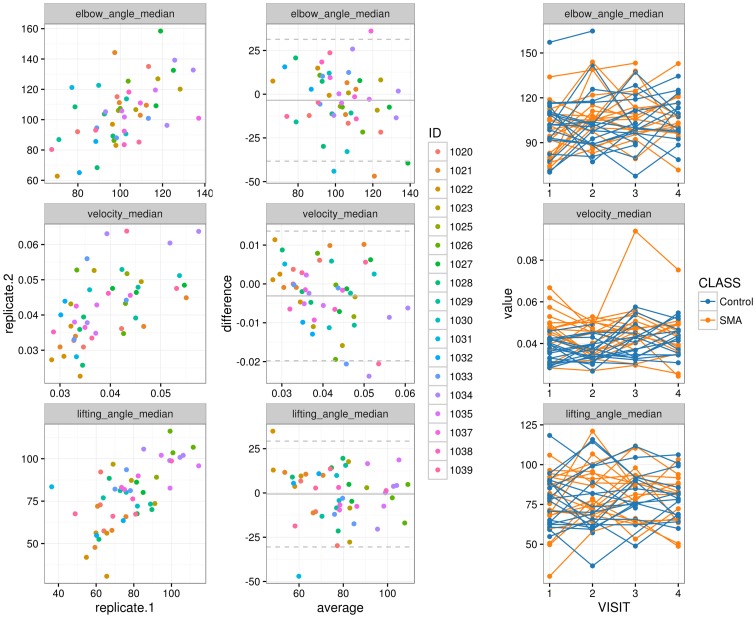
Repeatability. The first column shows the scatter plots of three features for the two assessments of controls within the same day (angles in degrees and velocity in m/s). The second column shows the Bland-Altman plots of the same two assessments. Values are colored by individual IDs of the controls. The third column displays between visit assessments for SMA patients and controls. Measurements from the same subject are connected by lines and are colored by groups.

Fig 3 shows the analysis for repeatability. For elbow angle, lifting angle and hand velocity, the scatter plots (first column) illustrate within day correlation of assessments for the controls between test and retest. The Bland-Altman plots (second column) show the difference against the average of the two assessments with mean and two times standard deviation range. The third column shows the first assessment per day across all visits for patients and controls. Each line connects the measurements of one subject.

We repeated the correlation analysis for between visit data on the patient and the control population. Within day correlation of replicates for controls and average pairwise correlation between visits for patients and controls are listed below for the three features (Table 2).

**Table 2:** Pearson correlations for the three features from test-retest data within the same day for controls and from between visits for controls and patients.

variable	SMA_Between	Control_Between	Ctrl_Intraday
elbow_angle_median	0.33	0.38	0.55
lifting_angle_median	0.54	0.68	0.64
velocity_median	0.45	0.27	0.58

**Fig 4:**
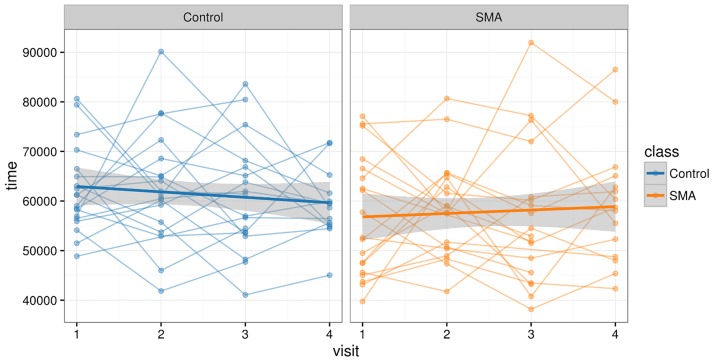
Learning effect. Total time spent in finishing the test for all visits is plotted with lines connecting the records from the same subject. Thick lines display the linear fit per group, with 95% confidence intervals.

In Fig 4 for all visits the total time needed is displayed in milliseconds. Thin lines connect the records for the same subject. A repeated measures ANOVA on the assessment times cannot reveal any significant time effect (p = 0.7728). Subjects do not gain in speed, which is an indicator for the absence of a learning effect.

### Comparison of patients and controls

The PCA was repeated on the data set without the 2 outliers. The first principal components do not separate the patients from the controls and there are also no individual features driving the loadings of the first 5 principal components (see S1 Text).

**Fig 5:**
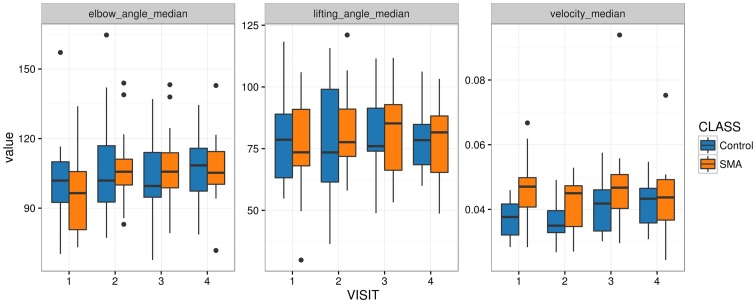
Feature—disease association. Distributions of three features are displayed by group and by visit. Elbow angle and lifting angle show no group differences as opposed to velocity.

Since a multivariate approach did not lead to group separation, each feature was tested for a difference between patients and controls individually, using a mixed effects model (see Methods, Statistical Analysis). After FDR adjustment only one feature showed a significant alteration (p<0.05) between the two subject groups, the median velocity of the hand (Fig 5).

## Discussion

In this study we have demonstrated the applicability of the Microsoft Kinect sensor to address the critical need for more objective and sensitive outcome measures in spinal muscular atrophy. With a specifically designed and user-friendly game, upper limb movement was captured markerlessly through time resolved body point location, which allowed detailed analysis of joint motion limitations.

The whole setup, operation and data handling were carried out smoothly. Only one half an hour training at the beginning for the operators was necessary. Therefore, this platform has the potential to give reliable and objective information as an essential complement to functional rating scales scored by trained clinicians. During the study, two small practical issues needed to be addressed. It was observed that in some cases patients were not detected very well by the infrared Kinect camera due to the colour or texture of clothes which still needs to be further investigated. This also happened when the infrared beam interfered with strong sunshine. Additionally, the software expects only one participant. However, if another person stays within the recording area, tracking may jump between the two. This situation happened once but the respective record was identified as an outlier in the PCA and the issue was revealed by an animated replay as in S1 Video of the data.

Two features of main interest, elbow angle and arm lifting angle, did not show any difference in SMA type III patients compared to healthy controls, which is consistent with previous literature regarding joint limitations [30]. By using transparent goniometers to measure joint range of motion of the shoulder, elbow, wrist, hip, knee and ankle, it has been found that joint limitations among SMA type III were less common. Unfortunately, our initial intention to detect early stage progression or minor difference in those high functioning patients by detailed tracking of active upper limb movement is not achieved by the current design. Whether the game can reveal differences in type II patients remains to be tested. In addition, Upper Limb Module [25, 33] which was developed to increase the range of assessment and eliminate the ceiling effect in SMA type III population might be a good reference for validation in the future.

The hand velocity was found to discriminate SMA patients and healthy controls, in that the SMA patients were slightly faster. This could emerge from different scenarios. In this game a fast speed was not required, therefore differences in velocity could result from the motivation. To validate this finding, a follow-up task, dedicated to velocity measures will have to be developed. One important and common indication related to velocity in these patient population is fatigue [5, 34–36]. Whether fatigue over time can be captured by repeated movements is to be considered for future software and study design.

The current work was a pilot study, and the development of this technology is a dynamic and interactive process as the more we know about patients’ limitation the better the game can be designed. One obstacle for the development of better clinical endpoints for SMA is that the disease is rare. There are not many trials and thus it is difficult to sufficiently validate new technologies.

In conclusion this study suggests that the Microsoft Kinect sensor has the potential of being developed into a complementary output measure for spinal muscular atrophy as it provides reproducible, objective and detailed information of body point motion. The data interpretation is clear and straightforward since it directly records the body points’ spatial locations. The current game design is not sensitive enough to capture the minor differences or early stage progression in high functioning patient group. Further adaptation and measure selection based on a better understanding of patients’ capabilities are essential to demonstrate the potential capability of this new technology.

## Supporting Information

S1 Text Complete analysis report generated by R. The whole analysis report including additional figures, tables and statistical tools as supplemental material to the main content of this publication is provided in this file.
(PDF)

S1 Code Source code from prototype game to data analysis. (ZIP)

S1 Dataset Related data files including raw data files, extracted feature file and demographics. (ZIP)

S1 Video An animated replay video of one outlier data file. (MP4)
