# Supplementary material for: Feasibility of Using Microsoft Kinect to Assess Upper Limb Movement in Type III Spinal Muscular Atrophy Patients
Source: PLoS One. 2017 Jan 25;12(1):e0170472. doi: 10.1371/journal.pone.0170472 (PMC5266257; doi:10.1371/journal.pone.0170472)
Supplement: S1 Text — The whole analysis report including additional figures, tables and statistical tools as supplemental material to the main content of this publication is provided in this file. (PDF) [file pone.0170472.s001.pdf]

Supplemental material

Xing Chen, Juliane Siebourg-Polster, Detlef Wolf, Christian Czech, Ulrike Bonati, Dirk Fischer, Omar Khwaja, Martin Strahm

- Data Overview
- Figure 2: Trace plot
- Outliers
- List of all features
- PCA plots
- Figure 3: Repeatability
- Figure 4: Learning effects
- Figure 5: Feature - disease association
- Repeated measures modelling on the Kinect features for class association
- Correlation for all features
- Animated replay of one subject’s movement during the task

Data Overview

Data were pre-processed and formatted using the script 1_dataProcessing.R (1_dataProcessing.R)

There are

- 37 individuals
- 18 patients
- 19 controls
- and a total of 187 observations
- from 134 unique visit days

Furthermore the per visit observations are

	1	2	3	4
Control	19	17	17	15
SMA	18	17	17	14

Number of

observations per
visit.

Figure 2: Trace plot

Movement trajectories of all 9 tracked body points in x-y dimension for a patient with a tremor and a healthy control.

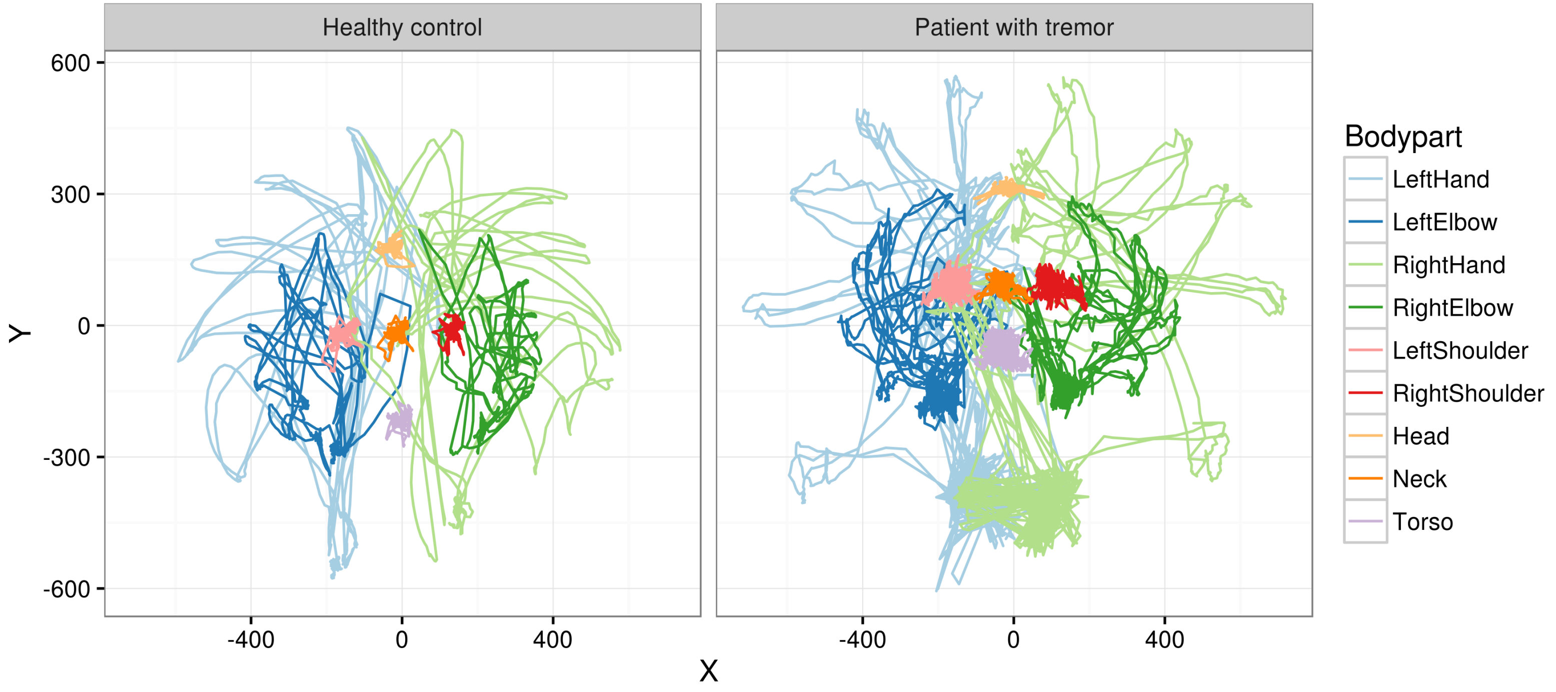

Outliers

The two outliers identified in the first component of a PCA on the full data set (see PCA plots, panel 1).

	ID	VISIT	REP	DATE	TIME	CLASS	elbow_angle_mean
1024_4_1	1024	4	1	2015-03-19	19.19	Control	129.8594
1038_3_1	1038	3	1	2014-12-11	17.45	Control	108.8756

List of all features

The final list of features, filtered for redundant or constant features. The name of each feature consists of the recorded measurement name and the summary statistic calculated from it. These are median, standard deviation, min and max values as well as various histogram sections (indicated with num_ range). The number of remaining features is 34.

elbow_angle_std, elbow_angle_median, elbow_angle_num_0.60, elbow_angle_num_60.120, elbow_angle_num_12.180, velocity_std, velocity_median, acceleration_pos_median, acceleration_neg_median, acceleration_num_.10, acceleration_num_.10..5, acceleration_num_.5.5, acceleration_num_5.10, acceleration_num_10., hand_path_length, hand.shoulder_max, hand.shoulder_min, hand.shoulder_std, hand.shoulder_median, hand.shoulder_num_0.0.4, hand.shoulder_num_0.4.0.8, hand.shoulder_num_0.8.1.2, lifting_angle_std, lifting_angle_median, lifting_angle_num_0.60, lifting_angle_num_60.120, lifting_angle_num_120.180, trunk_max, trunk_std, trunk_median, trunk_num_0.0.002, trunk_num_0.002.0.005, trunk_num_0.005., reachable_area

PCA plots

Panel 1 shows the two outliers identified in the first component of a PCA on the full data set. After filtering for outliers and uninformative features, a second PCA is performed to identify overall patterns in the Kinect features. Panel 2 shows a biplot of the first two PCs, coloured by the subject classes. Panel 3 shows the percentage of variance explained by the first 10 PCs and Panel 4 the loadings of the first 5 PCs. Here features are represented by numbers on the x axis.

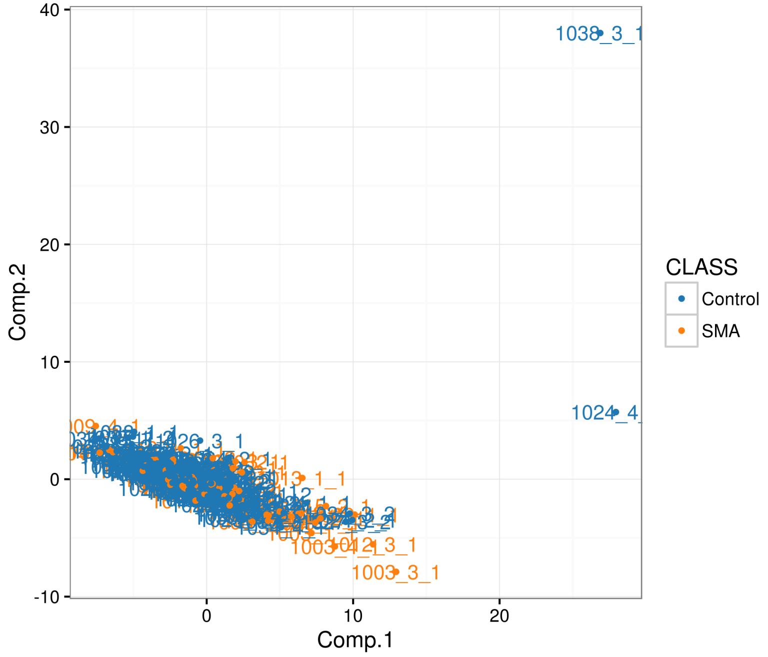
Comp.2
Comp.1
40
30
20
10
0
-10
0
10
20
CLASS
Control
SMA

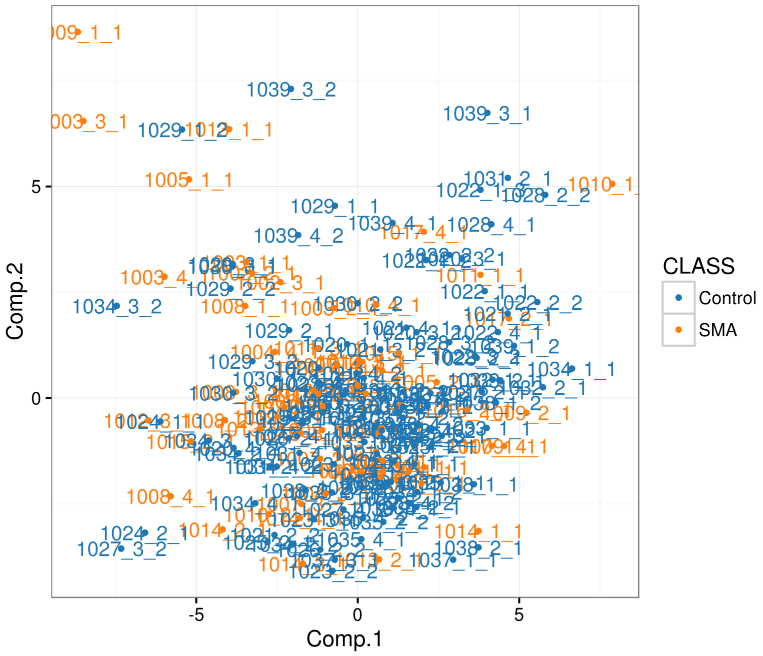
Comp.2
Comp.1
5
0
-5
0
5
CLASS
Control
SMA

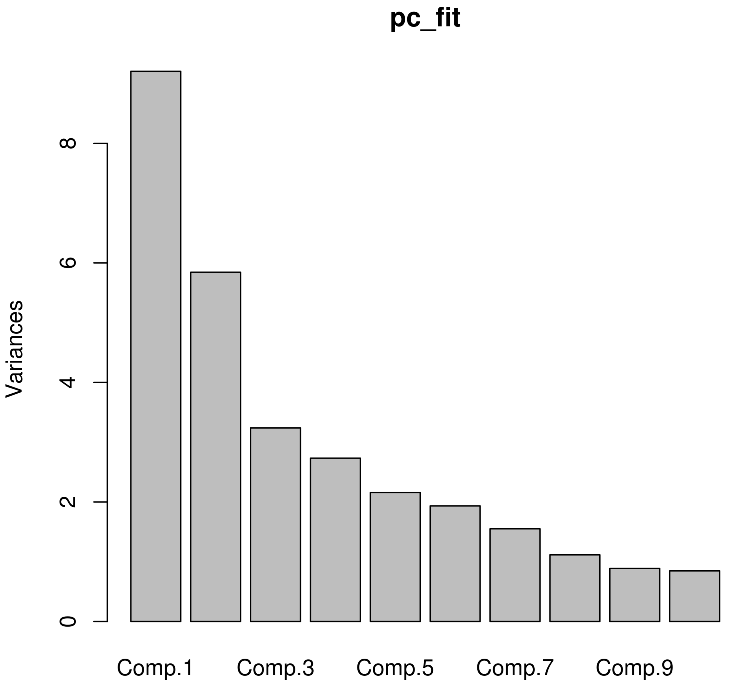
pc_fit
Variances
0
2
4
6
8
Comp.1
Comp.3
Comp.5
Comp.7
Comp.9

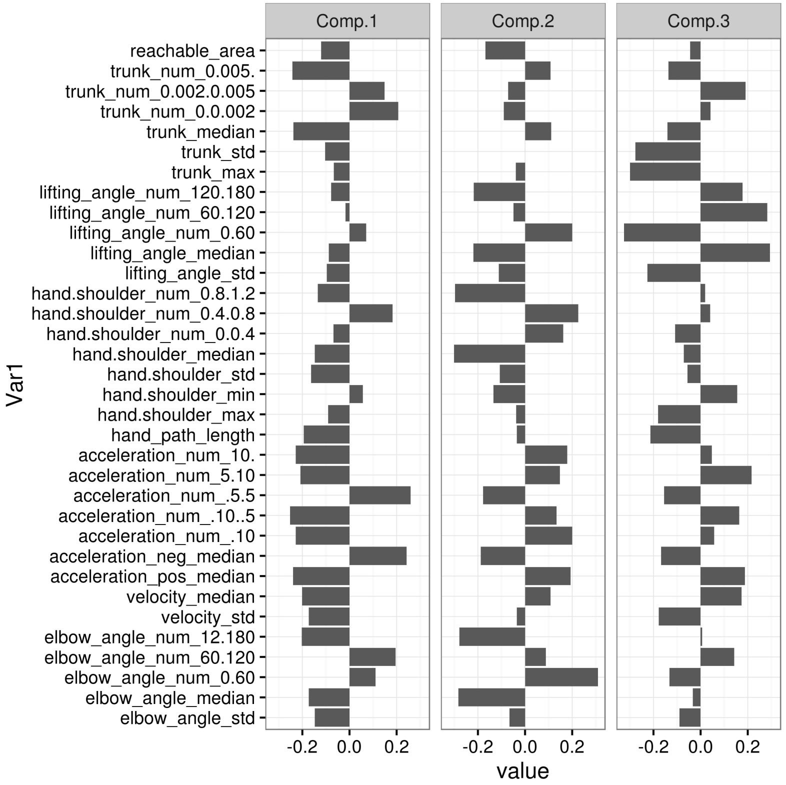

Figure 3: Repeatability

For elbow angle, lifting angle and velocity, the scatter plots (first column) illustrate within day correlation of assessments for the controls. The Bland-Altman plots (second column) show the difference against the average of the two assessments with mean and two times standard deviation range. The third column shows the first assessment per day across all visits for patients and controls. Each line connects the measurements of one subject.

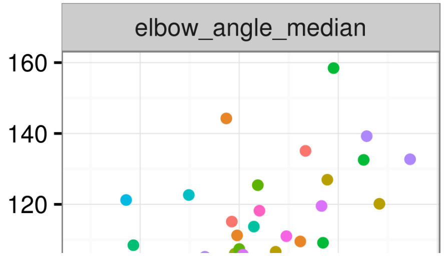

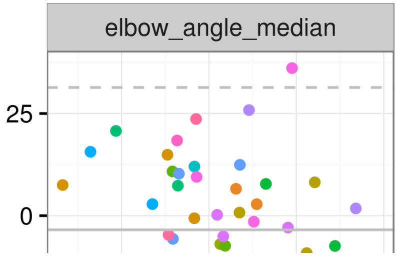

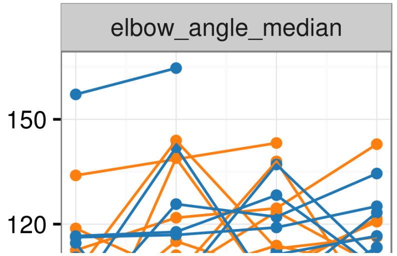

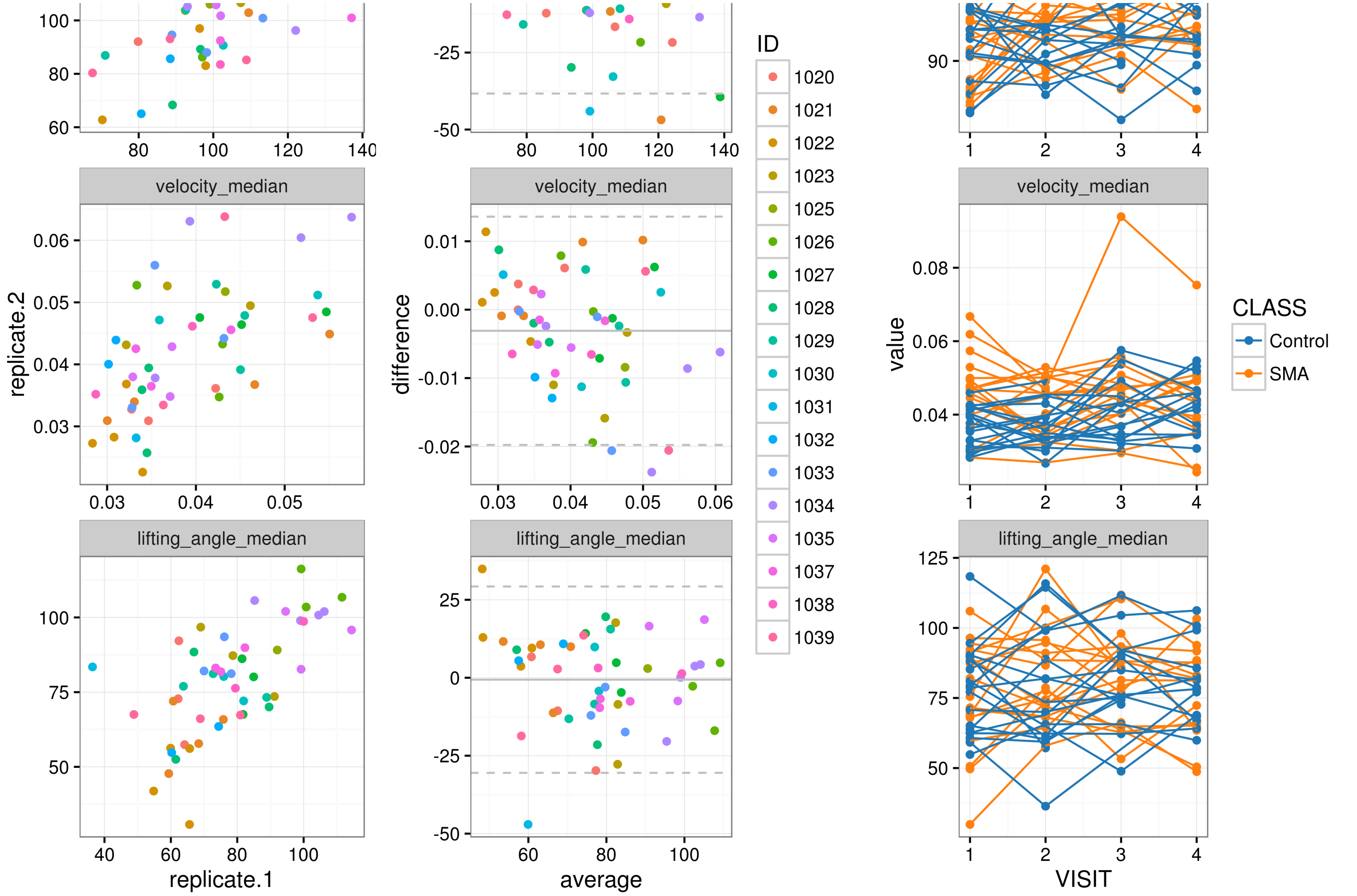

Within day correlation of replicates for controls and average pairwise correlation between visits for patients and controls are listed below for the three features.

variable	SMA_Between	Control_Between	Ctrl_Intraday

elbow_angle_median	0.33	0.38	0.55
lifting_angle_median	0.54	0.68	0.64
velocity_median	0.45	0.27	0.58

Figure 4: Learning effects

For all visits the total time needed is displayed in seconds. Thin lines connect the records for one subject. Thick lines display the linear fit per group, with confidence intervals.

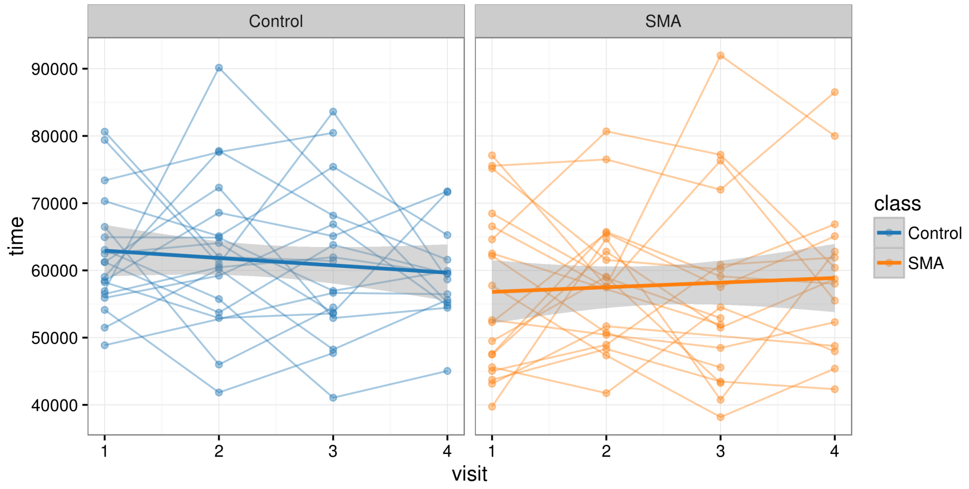

A repeated measures Anova on the assessment times cannot reveal any significant time effect. Subjects do not gain in speed, which is an indicator for the absence of a learning effect.

	numDF	denDF	F-value	p-value
(Intercept)	1	98	2043.7006166	0.0000000
factor(visit)	3	98	0.3727926	0.7728057
class	1	36	2.1758921	0.1488811

Figure 5: Feature - disease association

Distributions of three features are displayed by group and by visit. Elbow angle and lifting angle show no group differences as opposed to velocity.

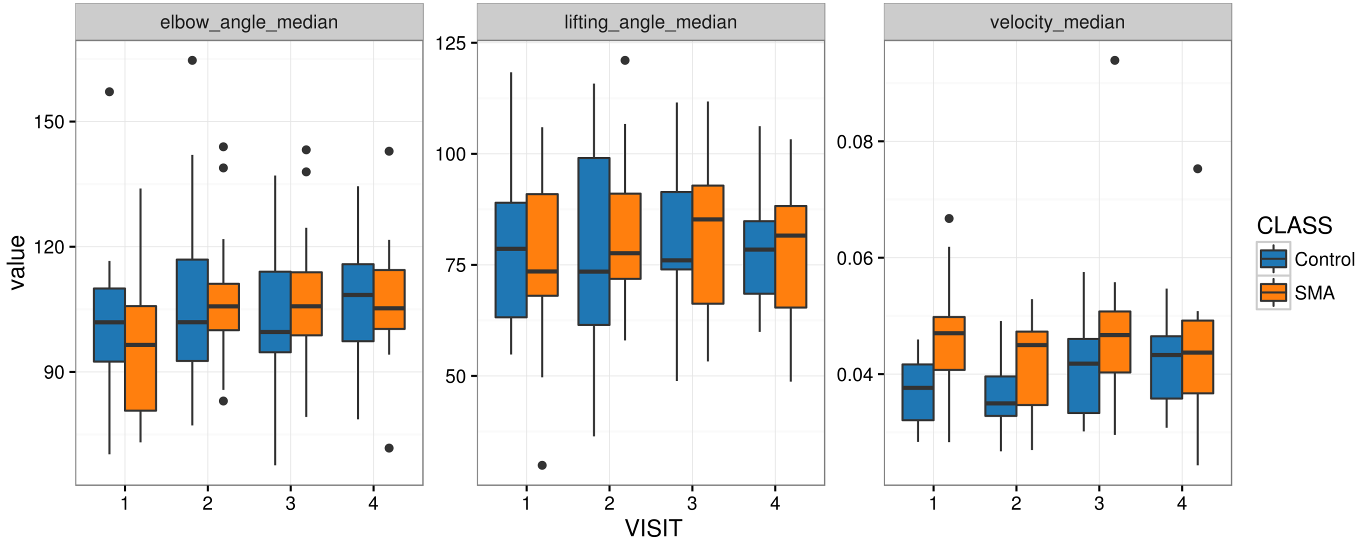

Repeated measures modelling on the Kinect features for class association

A linear mixed effects model is fit for each KINECT feature with CLASS (SMA / Control) as main predictor, using age, sex, BMI and height as covariates.

Only one measurement per day is used for each of the controls. It is fit for all 34 features.

The sample numbers are

	1	2	3	4
Control	19	17	17	14
SMA	18	17	17	14

In the following,

- data are scaled (z-scored), to compare effects of metabolites

- significance of all reported results, is based on FDR corrected p-values, is coded as:

```
0 < '***' < 0.001 < '**' < 0.01 < '*' < 0.05 < ' ' < 1
```

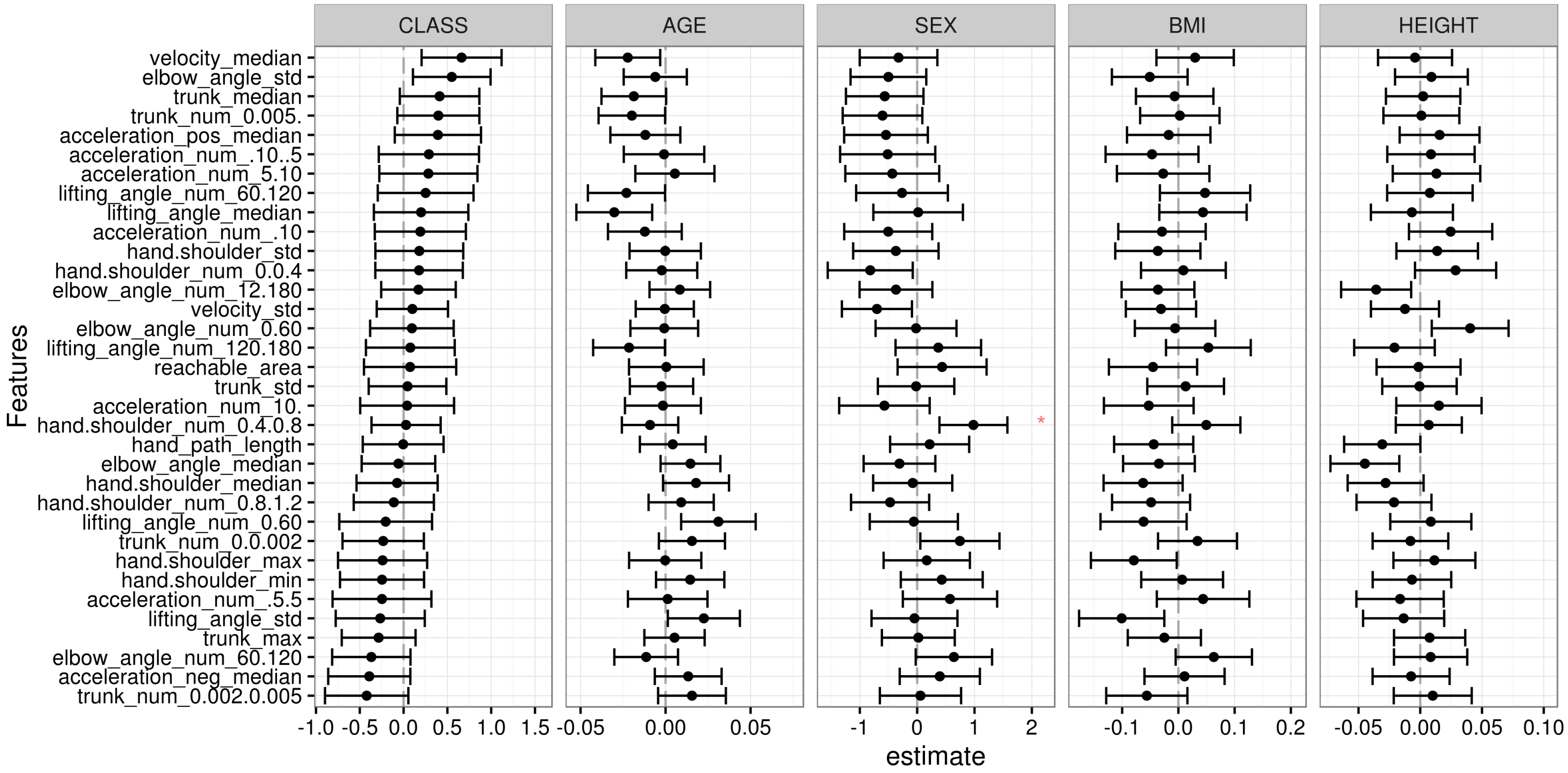

Velocity features show a trend towards higher values in patients. However, after FDR correction for 34 features, p values fall below the significance threshold of 0.05.

Top 3 features for class association

Each thin line denotes one subject. The thick lines represent the averages with standard deviation in grey.

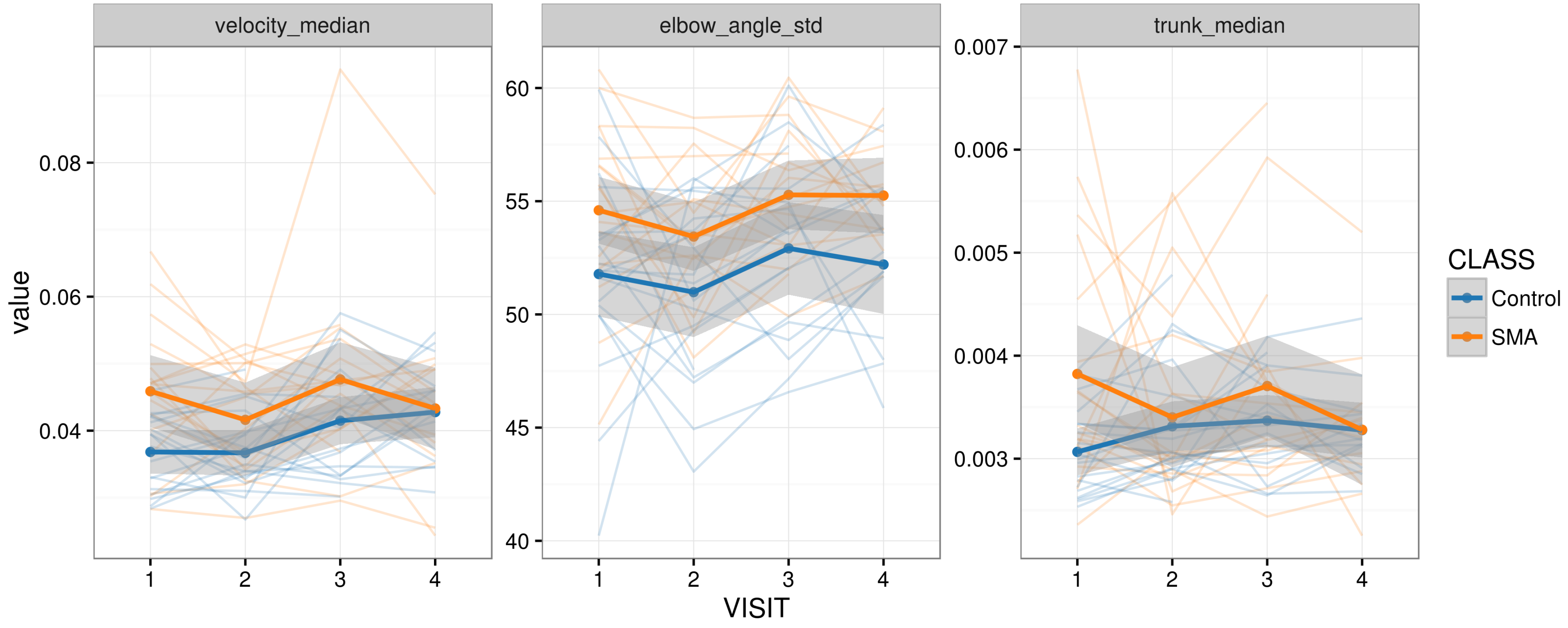

Correlation for all features

The distribution of correlation coefficients for the features are shown. The first box-plot shows the correlation of each feature for the two assessments of controls within the same day. The second box-plot shows the correlation of all features for controls between visit 1 and 2 and the third box-plot displays between visit correlation for SMA patients. Features are colored by their feature group as described in section 'Feature Extraction'.

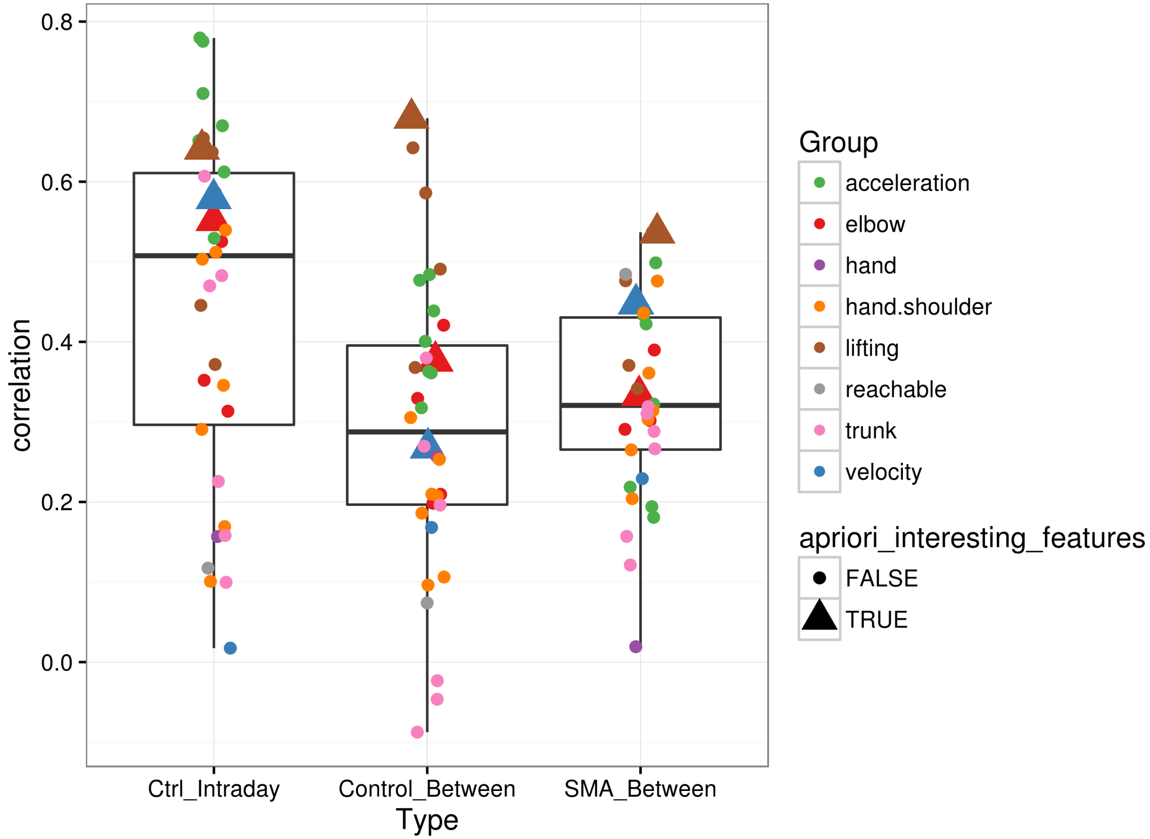

Animated replay of one subject’s movement during the task

The record was identified as outlier by PCA. Through animated replay it was found that the tracking jumped between the subject and another person in the room.

animated_replay.mp4 (../animated_replay.mp4)
